# Impairment in Extinction of Contextual and Cued Fear Following Post-Training Whole-Body Irradiation

**DOI:** 10.3389/fnbeh.2014.00231

**Published:** 2014-07-02

**Authors:** Reid H. J. Olsen, Tessa Marzulla, Jacob Raber

**Affiliations:** ^1^Department of Behavioral Neuroscience, Oregon Health and Science University, Portland, OR, USA; ^2^Division of Neuroscience, Oregon National Primate Research Center, Oregon Health and Science University, Portland, OR, USA; ^3^Department of Neurology, Oregon Health and Science University, Portland, OR, USA; ^4^Department of Radiation Medicine, Oregon Health and Science University, Portland, OR, USA

**Keywords:** irradiation, post-training, fear conditioning, wild-type mice, body weight, anxiety

## Abstract

Because of the use of radiation in cancer therapy, the risk of nuclear contamination from power plants, military conflicts, and terrorism, there is a compelling scientific and public health interest in the effects of environmental radiation exposure on brain function, in particular hippocampal function and learning and memory. Previous studies have emphasized changes in learning and memory following radiation exposure. These approaches have ignored the question of how radiation exposure might impact recently acquired memories, which might be acquired under traumatic circumstances (cancer treatment, nuclear disaster, etc.). To address the question of how radiation exposure might affect the processing and recall of recently acquired memories, we employed a fear conditioning paradigm wherein animals were trained, and subsequently irradiated (whole-body X-ray irradiation) 24 h later. Animals were given 2 weeks to recover, and were tested for retention and extinction of hippocampus-dependent contextual fear conditioning or hippocampus-independent cued fear conditioning. Exposure to irradiation following training was associated with reduced daily increases in body weights over the 22-days of the study and resulted in greater freezing levels and aberrant extinction 2 weeks later. This was also observed when the intensity of the training protocol was increased. Cued freezing levels and measures of anxiety 2 weeks after training were also higher in irradiated than sham-irradiated mice. In contrast to contextual freezing levels, cued freezing levels were even higher in irradiated mice receiving 5 shocks during training than sham-irradiated mice receiving 10 shocks during training. In addition, the effects of radiation on extinction of contextual fear were more profound than those on the extinction of cued fear. Thus, whole-body irradiation elevates contextual and cued fear memory recall.

## Introduction

Environmental whole-body exposure to radiation might occur as part of a natural disaster, an accident at a nuclear facility, a military nuclear conflict, or radiological terrorism. Coupled with an increasing interest in long-distance space-travel, as well as the use of radiation in cancer therapy, there is a compelling scientific and public health interest in the effect of radiation on brain function. One particular outcome of relevance is learning and memory. Most efforts to study the effects of radiation on this process have utilized paradigms, wherein animals are irradiated well before learning or memory testing with interesting results (Rosi et al., 2012; Allen et al., 2014). Less is known about earlier radiation effects on the brain. Novel object recognition 10 min following training was impaired in mice irradiated with 2 Gy prior to training but not in those irradiated with 5 or 8 Gy (Kumar et al., 2013). Diffusion tension imaging (DTI) performed 48 h following irradiation showed that the hippocampus and frontal cortex were especially sensitive to reduced fractional anisotropy, supporting hippocampal sensitivity to radiation. The sensitivity of the hippocampus to early gamma radiation effects is consistent with other radiation DTI studies (Trivedi et al., 2012). The reduction in myoinositol and taurine ratios in the cortical–hippocampus region 2–10 days after whole-body X-ray irradiation (8 Gy) in young adult mice using *in vivo* proton nuclear magnetic resonance spectroscopy (MRS) suggests perturbations in astrocytes or microglial activation (Rana et al., 2013). The specific involvement of the hippocampus is further supported by the recently reported memory preservation at 4 and 6 months follow up in patients with brain metastases receiving intensity-modulated radiotherapy to reduce exposure to the hippocampus (Gondi et al., 2012).

Microtubule-associated protein 2 (MAP-2) is important for the assembly of microtubules, particularly in the dendritic arbor, and is associated with changes in learning and memory (Harada et al., 2002). Following brain only ^56^Fe irradiation (600 MeV, 3 Gy) of 6–9-month-old mice, MAP-2 levels in the dentate gyrus were increased (Villasana et al., 2013). This might be a compensatory change as increased MAP-2 levels are also seen in the hippocampus and prefrontal cortex of aged non-human primates (Haley et al., 2010) and brains of aged mice (Benice et al., 2006). MAP-2 might also be altered within 2 weeks following irradiation.

Hippocampal sensitivity to radiation-induced cognitive injury is not limited to gamma irradiation and is also seen 2 weeks (Haley et al., 2012, 2013) or later (Shukitt-Hale et al., 2000; Villasana et al., 2010, 2011; Raber et al., 2011; Yeiser et al., 2013) following ^56^Fe irradiation. In all these studies, the animals were trained and tested for hippocampal function following irradiation. Due to adaptation following irradiation, other brain areas might compensate for brain areas most sensitive to irradiation. We designed a study to investigate the effects of radiation on previously acquired memories, which would not be processed or consolidated by some compensatory process. Therefore, in the current study mice were irradiated with X-rays 24 h following training and tested 2 weeks later for retention and extinction of hippocampus-dependent contextual fear conditioning. To assess whether such effects are limited to hippocampal function, an independent group of mice was tested for amygdala-dependent and hippocampus-independent memory as well as extinction of cued fear conditioning and measures of anxiety in the elevated zero maze.

Markers for hippocampal function, such as MAP-2 are altered during learning and memory tasks (Harada et al., 2002), as well as following a ^56^Fe radiation exposure (600 MeV, 3 Gy) in 6–9-month-old mice (Villasana et al., 2013). Therefore, effects on MAP-2 levels in the hippocampus of the mice were also analyzed by western blot.

## Mice

One-month-old male C57Bl6/J wild-type mice purchased from the Jackson Laboratory (Bar Harbor, ME, USA) were used for the current study. The mice were housed under a constant 12 h light:12 h dark cycle. Food (PicoLab Rodent Diet 20, no. 5053; PMI Nutrition International, St. Louis, MO, USA) and water were provided *ad libitum*. As the mice were 1-month old at the time of training and irradiation and tested 2 weeks later, they were 1.5-month-old at beginning of extinction. All procedures conformed to the relevant regulatory standards and were approved by Institutional Animal Care and Use Committee at Oregon Health and Science University (OHSU, Portland, OR, USA).

## Contextual Fear Conditioning

### Experimental groups

For all experiments, mice were assigned to experimental group (irradiated or non-irradiated) by repeated random sorting until all initial variables were equal between the groups. After fear conditioning training, and prior to irradiation, mice were randomly sorted until all initial values (body weight, baseline freezing, freezing levels after acquisition, etc.) were not significantly different between groups.

#### Experiment 1

Twenty mice were trained in a contextual fear conditioning paradigm, involving five 2-s 0.35 mA shocks, separated by 2-min inter-shock-intervals (ISI), with the first shock at 2 min from the beginning of the trail. The total length of the training session was 10 min. Twenty-four hours after training, all mice were brought to a room within the animal facility containing an X-ray irradiator (Rad Source RS2000 Biological Research Irradiator, Suwanee, GA, USA) for whole-body irradiation exposure. Half of the mice (irradiation group) were placed in a new mouse cage fitting in the irradiator and received whole-body irradiation at a dose of 4 Gy (dose rate: 1.25 Gy/min). The other half of the mice was placed in a new mouse cage and received a sham-irradiation procedure by being placed into a new cage, in a similarly confined and dark space, for the same duration of time. Fourteen days after training (or 13 days after irradiation or sham-irradiation), the mice were tested for recall and extinction of conditioned fear, over a period of 8 days. On day 9, the mice received a minimal reinstatement session: after a 2-min baseline period, one 2-s 0.35 mA shock was delivered. The mice remained in the testing chamber for an additional 8 min to maintain the same 10 min trial length in all trials. All freezing data in this paper were analyzed using Med Associates software. The software analyses freezing based on a proprietary algorithm scoring with freezing defined as no movement except respiration. The next day (day 10), recall of post-reinstatement hippocampus-dependent contextual fear recall was assessed by exposure to the training context. Mice were weighed the day after training (before irradiation), and every 3 days thereafter – for a total of eight measurements over the duration of the experiment.

#### Experiment 2

In order to control for differences in initial freezing to the context on day 14 (extinction trial 1) affecting extinction curves, Experiment 1 was repeated with another 20 mice, as described above with the following two exceptions. A 10 shock rather than a 5 shock paradigm was used during training and the shocks were separated by a 60-s ISI rather than a 120-s ISI. The pre-exposure period was therefore 60-s. The irradiation, sham-irradiation, and other experimental conditions of contextual fear conditioning testing were as described above in Experiment 1. The intent of this experiment was to ascertain whether differences in extinction could be due to reduced recall in one group compared to the other.

#### Experiment 3

To evaluate the contribution of non-hippocampus-dependent memory processes, 20 male mice were trained using a cued fear conditioning paradigm consisting of 5 shocks. A 60-s habituation period was followed by 30-s tones (2800 Hz, 80 dB) co-terminating with 2-s 0.35 mA shocks, and separated by 2-min ISI, and a final 2-min post-shock acquisition period. Twenty-four hours after training, mice were irradiated with 4 Gy or sham-irradiated as described in Experiment 1. Two weeks (14 days) after training (13 days after irradiation), the mice were tested for recall and extinction of cued fear over 8 days. Cued extinction trials consisted of the mouse being placed into an environment distinct from the one used during training (rounded walls, novel floor texture, cleaning with a 10% isopropanol solution). A 60-s baseline/habituation period was followed by five 60-s tone presentations separated by 60-s inter-stimulus-intervals. Mice were weighed the day after training and every 3 days thereafter.

#### Experiment 4

As in Experiment 2, in order to ascertain whether differences in extinction could be due to reduced recall in one group compared to the other, the cued experiment was repeated using 10 shocks, with the shocks separated by a 60-s ISI, and keeping all other experimental conditions as described as in Experiment 3.

#### Experiment 5

##### Elevated zero maze

To determine whether potential differences in measures of anxiety might contribute to altered performance in fear conditioning tests, mice from Experiments 3 and 4 were tested for anxiety-like phenotype in the elevated zero maze. Because the potential anxiety phenotype in question required a temporal proximity to the fear conditioning extinction testing, mice were tested 12 days after irradiation, 1 day before the beginning of the extinction experiment. To assess the impact of irradiation on anxiety-like phenotypes in the absence of exposure to a fear-inducing event, a group of 20-animals who did not receive fear conditioning, and only received radiation treatment or were sham-irradiated, were also evaluated in the zero maze for anxiety-like behaviors.

The elevated zero maze (Kinder Scientific, Poway, CA, USA) consisted of four sections (6 cm wide), alternating between open and closed sections. Mice were placed into an open area of the maze and allowed to explore the maze for 10 min. Mice treated with a wide-range of anxiety reducing agents spend less time in the open areas. An automated photo beam detection method (Kinder Motor Monitor software, Kinder Scientific, Poway, CA, USA) was used to track mouse movements. Outcome measures were distance moved (centimeter), time spent in the open and closed areas (second) as well as crossings between the open and closed areas.

*Western blot analysis*. Mice from Experiments 1 and 2 were killed by cervical dislocation and their brains removed. The hippocampi were dissected and immediately frozen in liquid nitrogen for western blot analysis. The hippocampi were homogenized separately in 1 ml or 300 μl of RIPA buffer (Pierce Pharmaceuticals, Rockford, IL, USA) containing 10% halt protease inhibitor cocktail (Pierce). Homogenized tissue was spun at 12,000 ×* g* for 15 min, and protein concentrations were determined in the supernatant using Pearce BCA protein assays (Pierce Pharmaceuticals, Rockford, IL, USA). The samples were stored at −80°C until use.

Proteins were denatured by boiling for 5 min at 99°C in a solution of Laemmli’s buffer containing 5% 2-mercaptoethanol (Bio-Rad, Hercules, CA, USA). For each sample, 40 μg of protein was loaded in a lane of pre-prepared gels (Criterion Bio-Rad Ready Gels, 4–15% Tris–HCl, 18 well). For each gel, one lane was loaded with Kaleidoscope™ Prestained Standards (Bio-Rad). The gels were placed in an electrophoresis apparatus and run with a Bio-Rad Power Pac for 60 min at 120 V. Proteins were transferred to PVDF membranes for 90 min at 100 V.

Once proteins were transferred to the membranes, the membranes were placed in 3% bovine serum albumin (BSA) Tris buffered saline containing 0.5% Tween (TBST) blocking buffer for 1 h. Membranes were washed in TBST buffer (4× for 5 min) and incubated in 3% BSA TBST with one of the following primary antibodies for 12 h at 4°C: antibodies against MAP-2 (raised in mouse, 1 μg/ml, Millipore, Billerica, MA, USA) or β-actin antibody (raised in mouse, 0.5 μg/ml, Santa Cruz Biotechnology, Santa Cruz, CA, USA). There were no effects of irradiation on β-actin levels. Membranes were washed in the TBST buffer (4× for 5 min) and were incubated in secondary antibody (Santa Cruz, goat anti-mouse-HRP, 1 μg/ml) in the 3% BSA TBST buffer for 1 h. Membranes were incubated in SuperSignal West Pico solution (Bio-Rad) for 5 min and pixel densities of specific bands of MAP-2 and β-actin for each sample were imaged and quantified using densitometry with Image Lab software (Image Lab™ Software, Bio-Rad Laboratories, Inc., Hercules, CA, USA). Background levels were automatically determined by the software using upper- and lower-edge interpolation. The MAP-2 and β-actin bands were measured for each sample. Antibodies were stripped from the membranes using Restore Western Blot stripping buffer (Thermo Scientific) for 5 min at room temperature and re-blocked in 3% BSA TBST blocking buffer for 1 h. β-actin was used as a loading control for each membrane. Data were analyzed as a ratio between the MAP-2 and β-actin bands compared to sham-irradiated levels for a specific blot.

*Statistical analyses*. Analyses were conducted using SPSS 16.0 software (Chicago, IL, USA). Baseline measures between groups were analyzed by ANOVA, with treatment group as a between-subject variable. Comparisons of freezing, motion during shock, and body weight over time were performed as repeated measures ANOVA, with treatment as a between-subject variable and the time-unit as a within-subject variable. Data were evaluated as to their satisfaction of assumptions for parametric statistics. If the data were skewed or otherwise non-normally distributed or did not have equal variances, appropriate transformations were applied. For repeated measures analysis, if Mauchly’s test of sphericity was violated, multivariate statistics reporting Welch’s lambda (λ) were reported. For pairwise comparisons, Dunnet’s *post hoc* were performed to compare selected values (between days and between groups). In the case of a between-subject variable interaction with a within-subject variable, the between-subject groups were separately analyzed to evaluate the potential difference in within-subject effects being mediated by the between-subject variable.

## Results

### Contextual fear conditioning (Experiments 1 and 2)

#### Effects of irradiation on body growth

**Figure 1:**
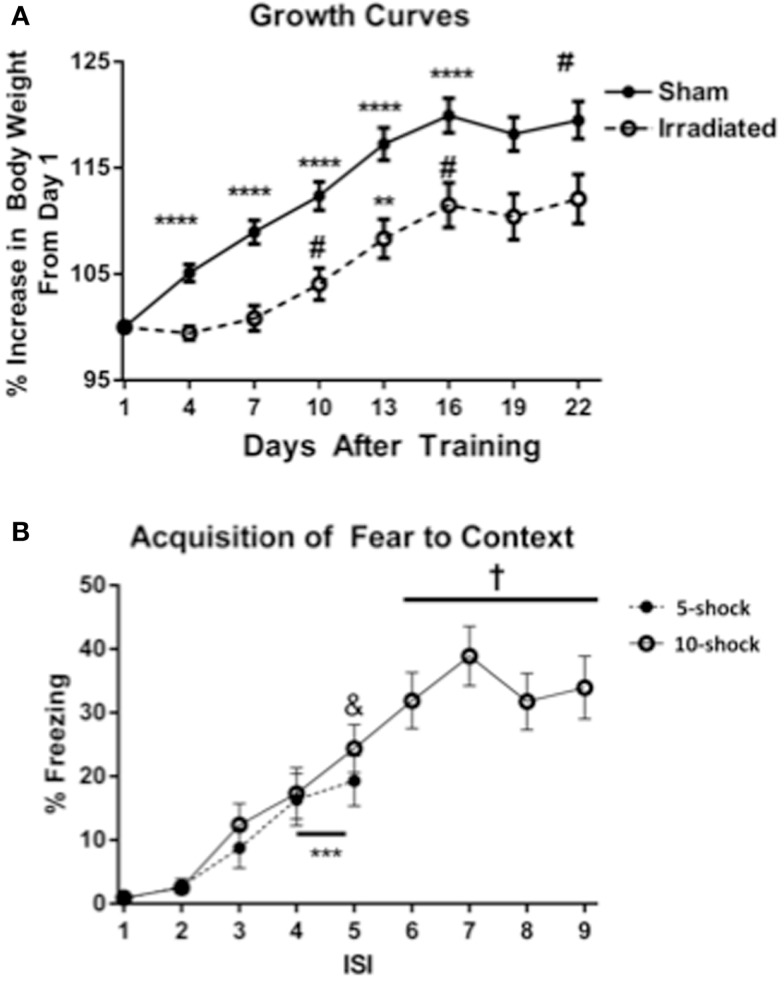
**(A)** Effects of irradiation on body growth in mice trained and tested for contextual fear conditioning. *p < 0.05, **p < 0.01, ***p < 0.001, ***p < 0.0001. **(B)** Acquisition of contextual fear conditioning, analyzed as immediate freezing during the ISI following a shock. ***p = 0.002 (vs. ISI 3), ^&^*p* = 0.025 vs. ISI 3, ^+^*p* < 0.0001 vs. ISI’s 3, 4, and 5.

Body weights of animals from Experiments 1 and 2 (5 and 10 shock) were analyzed together, with experimental group as a between-subjects variable. Body weight at the beginning of the experiment did not differ between treatment groups. Over the course of the experiment, an effect of day on body weight was observed [λ = 0.086 *F*(7,30) = 45.420, *p* < 0.0001], as well as significant interactions with treatment [λ = 0.432, *F*(7,30) = 5.626, *p* < 0.0001]. There was also a between-subjects effect of treatment [*F*(7,30) = 13.443, *p* = 0.0008], showing that irradiated animals weighed less than sham-irradiated animals. This effect was observed across both context and cued groups (Figure 1A).

Both treatment groups (radiation and sham-irradiated) exhibited increases in body weight [sham-irradiated: λ = 0.066, *F*(7,13) = 26.361, *p* < 0.0001; irradiated: λ = 0.062 *F*(7,13) = 28.322, *p* < 0.0001]. However, the increases between the treatment groups were dissimilar: sham-irradiated mice exhibited increases between day 1 and most subsequent days (day 4 vs. 1 *p* < 0.0001; day 7 vs. 4 *p* = 0.0007; day 10 vs. 7 *p* = 0.0021; day 13 vs. 10 *p* < 0.0001; day 16 vs. 13 *p* = 0.0108). In contrast, irradiated mice exhibited step-wise increases in body weight by day 7 (between 7 and 10, *p* = 0.0229), an increase between 10 and 13 days (*p* = 0.0037), and between 13 and 16 days (*p* = 0.0250). Although both experimental groups experienced growth, the increases in body weight were therefore retarded in the irradiated group during the course of the experiment.

#### Effects of irradiation on recall of contextual fear

##### Baseline freezing

Baseline freezing in the contextual fear experiment refers to freezing before training, when the mice are exposed to the context for the first time. Baseline freezing was not different between treatment groups in either the 5 or 10 shock paradigm. Baseline freezing in response to context did not differ between experimental groups in either experiment. These data indicate that there were no initial differences in baseline freezing in the animals.

##### Average motion during the shocks

To determine potential pre-existing group differences in sensitivity or perception to the aversive stimuli, average motion during the shocks was analyzed. The average motion during the shocks did not differ between the experimental conditions (Tables S1 and S2 in Supplementary Material).

##### Acquisition of conditioned fear

Acquisition of conditioned fear was assessed as immediate freezing during the ISI following a shock. Because most values for freezing were zero for ISI 1 and 2, analysis was conducted using ISI time bins 3, 4, and 5. In Experiment 1 (five shock), there was an increase in freezing with increasing shock number [λ = 0.352, *F*(2,17) = 15.634, *p* < 0.0001], but no differences between groups. Freezing levels increased from ISI 3 to ISIs 4 and 5 (*p* = 0.002), though the increase between ISI 4 and 5 was insignificant. In Experiment 2 (10 shock), there was also an effect of increasing number of shocks [λ = 0.101, *F*(6,13) = 19.195, *p* < 0.0001], but no difference between groups. Freezing levels from ISI 5 were significantly greater than those from ISI 3 (ISI 5 vs. 3, *p* = 0.025), and those of ISIs 6, 7, 8, and 9 were also significantly greater than those from ISIs 3 (*p* < 0.0001) (Figure 1B).

##### Extinction

Our study focused on between-trial extinction. Therefore, we analyzed the first 5 min of the extinction trials, to avoid the influence of habituation.

**Figure 2:**
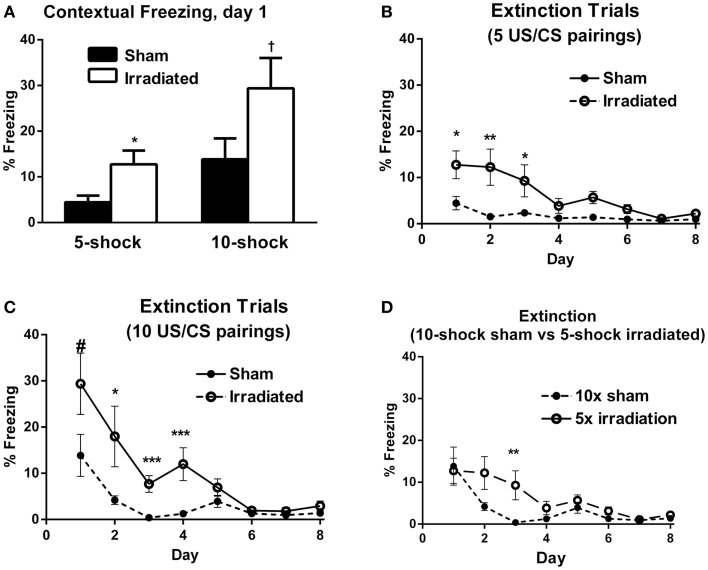
**(A)** Contextual freezing levels on day 1 (24 h after training) were higher in irradiated than sham-irradiated mice. *p = 0.021, ^+^*p* = 0.083. **(B)** Extinction of contextual fear conditioning in sham-irradiated and irradiated mice that received five tone-shock pairings (Experiment 1) during training. *p < 0.05, **p < 0.01. **(C)** Extinction of contextual fear conditioning in sham-irradiated and irradiated mice that received 10 tone-shock pairings (Experiment 2) during training. *p < 0.01, ***p < 0.0001, ^#^*p* = 0.083. **(D)** Extinction of contextual fear conditioning in sham-irradiated mice that received 10 tone-shock pairings during training and irradiated mice that received 5 tone-shock pairings during training. *p < 0.01.

In Experiment 1 (five shock), freezing levels on day 1 of the extinction trials were higher in the irradiated than sham-irradiated mice [Figure 2A; *F*(1,18) = 6.381, *p* = 0.021]. During extinction, there was a main effect of day [λ = 0.141, *F*(7,12) = 10.466, *p* < 0.0001], as well as an interaction with treatment [Figure 2B; λ = 0.365, *F*(7,12) = 2.980, *p* = 0.047], suggesting an effect of radiation on extinction of conditioned fear. A between-subject effect of radiation was also observed [*F*(1,18) = 6.689, *p* = 0.019], with irradiated mice exhibiting greater overall freezing than sham-irradiated mice.

Both groups exhibited extinction of conditioned contextual fear [sham-irradiated: *F*(7,63) = 3.504, *p* = 0.003; irradiated: *F*(7,63) = 11.013, *p* < 0.0001], but there were profound differences between irradiated and sham-irradiated animals. While both groups showed significantly reduced freezing relative to day 1 by day 4 (Figure 2B; Table S3 in Supplementary Material), irradiated mice exhibited persistently elevated freezing levels relative to the control group on days 1 (*p* = 0.021), 2 (*p* = 0.010), 5 (*p* = 0.003), and 6 (*p* = 0.0028) (Figure 2B).

In Experiment 2, irradiated mice exhibited a trend toward higher levels of freezing compared to their sham-irradiated counterparts on day 1 of extinction, but this did not reach significance [Figure 2A; *F*(1,18) = 3.370, *p* = 0.083]. Both groups showed gradual extinction of conditioned fear [Figure 2C; effect of day λ = 0.078, *F*(7,12) = 20.352, *p* < 0.0001]; however, this effect was modulated by treatment group [day × treatment interaction: λ = 0.357, *F*(7,12) = 3.082, *p* = 0.042]. A between-subject effect of radiation was also observed [*F*(1,18) = 13.760, *p* = 0.002], with irradiated mice exhibiting greater overall freezing than sham-irradiated mice, as in Experiment 1. While there was an effect of day on freezing levels for both sham-irradiated and irradiated mice [λ = 0.028, *F*(7,3) = 14.928, *p* = 0.024; *F*(7,63) = 15.459, *p* < 0.0001, respectively], extinction occurred less steeply in the irradiated group, which maintained elevated freezing relative to the sham-irradiated group on days 2 (*p* = 0.004), 3 (*p* < 0.0001), and 5 (*p* = 0.002) (Figure 2C).

Comparing initial contextual freezing in irradiated mice from Experiment 1 (5 shocks) to sham-irradiated mice from Experiment 2 (10 shocks) revealed significant parity [*F*(1,18) = 0.083, *p* = 0.777] between the groups (Figure 2D). Therefore, analysis of the extinction curves between these two groups was also conducted. There was a main effect of day [λ = 0.098, *F*(7,12) = 15.825, *p* < 0.0001] and a trend toward an interaction between the groups [λ = 0.395, *F*(7,12) = 2.625, *p* = 0.068]. While freezing levels were matched on the first day of extinction, extinction was retarded in irradiated animals that received fewer shocks (5) compared to sham-irradiated animals (10 shocks) (Figure 2D). So while the “strong-conditioning” sham group now froze comparable to the irradiated group with the reduced conditioning protocol, there was an effect of irradiation, with the irradiated group showing delayed onset of extinction. Overall, average freezing throughout extinction in the irradiated-5 shock group (6.27 ± 1.61%) was greater than the sham-irradiated-10 shock group (3.40 ± 1.57%), but this did not reach significance (*p* = 0.077).

##### Reinstatement

In Experiment 1, following reinstatement of the unconditioned stimulus, the sham-irradiated mice showed a trend toward an increase in freezing (*W*  = −35, *Z* = −1.38, *p* = 0.084), while the irradiated mice exhibited a significant increase in freezing (*W*  = −37, *Z* = −1.92, *p* = 0.0273). Additionally, freezing levels after the shock were greater in the irradiated group than in the sham-irradiated group (*t*_9_ = 2.853, *p* = 0.019) (Table S3 in Supplementary Material). Twenty-four hours after reinstatement (day 10), freezing levels in either group did not differ.

In Experiment 2, data from three mice were lost due to an equipment malfunction (two mice from the irradiated and one mouse from the sham-irradiated group). There was an increase in freezing after shock in both groups (sham-irradiated: *W*  = −28.00, *Z* = −2.15, *p* = 0.0156; irradiated: *W*  = −32.00, *Z* = −1.99, *p* = 0.0234). There was no difference in post-shock freezing between the groups. An analysis of freezing on the subsequent day (day 10) indicated a trend toward higher freezing in the irradiated group [*F*(1,15) = 3.370, *p* = 0.086] (Table S4 in Supplementary Material). This lack of significance may have been due to the slightly reduced sample size because of the equipment malfunctioning. Furthermore, mice who received 10 shocks but no irradiation performed at parity with those who received 5 shocks and irradiation. These data, taken with the results from Experiment 1, suggest that reinstatement of the CS–US relationship produced a stronger effect in the irradiated mice, which can be overcome with greater training (e.g., 10 vs. 5 shocks).

### Cued fear conditioning (Experiments 3 and 4)

#### Effects of irradiation on body growth

**Figure 3:**
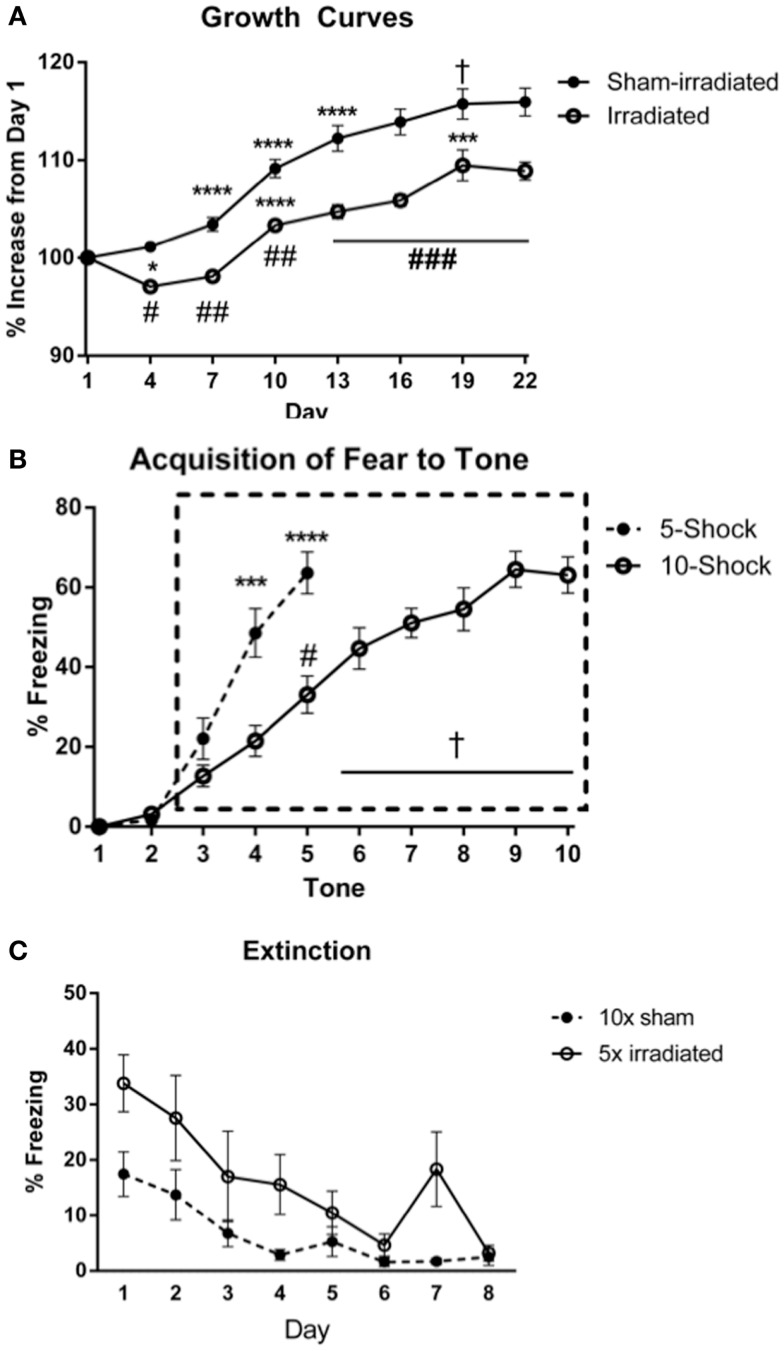
**(A)** Effects of irradiation on body weights of mice trained and tested for cued fear conditioning. *Comparisons between days, ^#^comparisons between groups. *p < 0.05, ***p < 0.001, ****p < 0.0001, ^#^*p* < 0.01 = , ^##^*p* < 0.0001, ^+^*p* = 0.090, ^&^*p* = 0.076. **(B)** Acquisition of fear to the tone. ****p < 0.0001, ^#^*p* = 0.005, ^+^*p* < 0.0001. Freezing levels during ISI 6–10 were significantly greater than those during ISI 3 (ISI 6 vs. 3, *p* = 0.007; ISIs 7, 8, 10 vs. 3, *p* < 0.0001; ISI 9 vs. 3, *p* = 0.001), and freezing during ISIs 7, 8, 9, 10 were higher than those during ISI 4 (ISI 7 vs. 4, *p* = 0.001, ISI 8 vs. 4, *p* = 0.004, ISI 9 vs. 4, *p* = 0.012, and ISI 10 vs. 4, *p* = 0.028), ISI 5 (ISI 7 vs. 5, *p* = 0.002, ISI 8 vs. 5, *p* < 0.0001, ISI 9 vs. 5, *p* = 0.03, and ISI 10 vs. 5, *p* = 0.020). **(C)** Comparison of freezing levels between irradiated and sham-irradiated animals during extinction of cued fear. *p < 0.05.

Body weights (Figure 3A) at the beginning of the experiment did not differ between treatment groups [Experiment 3 (5 shocks): sham: 18.82 ± 0.62 g; irradiated: 18.82 ± 0.45 g; Experiment 4 (10 shocks): sham: 18.99 ± 0.33 g; irradiation: 19.06 ± 0.16 g]. Body weight increased over time [λ = 0.052, *F*(7,30) = 78.016, *p* < 0.0001], and this was modulated by treatment group [λ = 0.460, *F*(7,30) = 5.037, *p* = 0.001]. There was also a between-subjects effect of treatment [*F*(1,36) = 27.096, *p* < 0.0001], which indicated that overall, irradiated animals weighed less than sham-irradiated animals. Sham-irradiated mice showed greater body weights than irradiated mice on each day following irradiation (All days *p* < 0.0001, except day 19, *p* = 0.007). Consistent with the results in Experiments 1 and 2, both groups experienced growth over time but increases in body weights were lower in the irradiated than sham-irradiated mice during the course of the experiment.

#### Effects of irradiation on cued fear conditioning

##### Baseline freezing

Baseline freezing prior to the first tone did not differ between treatment groups in either experiment.

##### Acquisition of conditioned fear

The average motion during the shocks did not differ between the groups. An effect of shock-order was indicated [*F*(4,72) = 6.047, *p* < 0.0001] with motion during shock 5 being statistically greater than shocks 1 (*p* = 0.003) and 2 (*p* = 0.007) (Table S5 in Supplementary Material). Acquisition of conditioned fear was assessed as immediate freezing during the ISI or tone following a shock. As in the prior fear conditioning experiments, freezing during the first two ISIs were not statistically different than zero, and thus analysis of ISI freezing was conducted starting at ISI 3 (Figure 3B). This was similarly the case for freezing during a tone following a shock.

#### Experiment 3 (5 shocks)

##### ISI

There was an effect of ISI [Figure 3B; *F*(2,36) = 6.332, *p* = 0.004], but no interaction with treatment group. Freezing levels were higher in ISI 3 than ISI 4 (*p* = 0.004) and ISI 5 (*p* = 0.008), though there was no significant increase from ISI 4 to 5.

##### Tone

Next, freezing during the tone was analyzed. There was an effect of tone order [*F*(2,36) = 51.789, *p* < 0.0001], but no interaction with treatment group. Freezing levels were higher during tones 4 and 5 than tone 3 (*p* < 0.0001), and during tone 5 than tone 4 (*p* < 0.0001).

#### Experiment 4 (10 shocks)

##### ISI

The average motion during the shocks did not differ between the groups. An effect of shock-order was not found (Table S6 in Supplementary Material). There was an effect of increasing ISI number [Figure 3B; *F*(7,126) = 7.017, *p* < 0.0001], but no interaction with treatment group.

##### Tone

All groups acquired fear to the tone. There was an effect of increasing tone presentations [*F*(7,126) = 29.591, *p* < 0.0001], but no interaction with treatment group. Freezing levels during tones 5–10 were significantly greater than those during tone 3 (tone 5 vs. 3, *p* = 0.005; tones 6, 7, 8, 9, 10 vs. 3, *p* < 0.0001), and freezing levels during tones 5, 6, 7, 8, 9, 10 were elevated compared to those during tone 4 (tone 6 vs. 4, *p* = 0.023; tones 7, 8, 9, and 10 vs. 4, *p* < 0.0001). Freezing levels during tones 7, 8, 9, 10 were elevated compared to those during tone 5 (tone 8 vs. 5, *p* = 0.040, tone 8 vs. 5, *p* = 0.001, tones 9 vs. 5, *p* < 0.0001, and tone 10 vs. 5, *p* = 0.001). Freezing levels during tones 9 (*p* = 0.004) and 10 (*p* = 0.003) were elevated compared to those during tone 6. Freezing during tones 9 (*p* < 0.0001) and 10 (*p* = 0.017) were also higher than freezing levels during tone 7.

##### Acquisition

No differences in acquisition were observed between groups. Interestingly, visual inspection of the acquisition of freezing to the tone shows showed a delayed slope in Experiment 4 (10 shocks). This may be due to the decreased length of the ISI in this experiment compared to that in Experiment 3. Comparing freezing between the paradigms during the ISI and tones, there was an interaction between tone and paradigm [*F*(2,76) = 7.542, *p* = 0.001], but not between ISI and paradigm. Mice trained in the 5 shock paradigm (Experiment 3) acquired fear to the tone at a faster rate than those in the 10 shock paradigm (Experiment 3; tone 4 vs. 3, *p* = 0.0003; Experiment 4, tone 5 vs. 3, *p* < 0.0001. Mice in the 5 shock paradigm also exhibited greater overall freezing during this period [*F*(1,38) = 14.987; 5 shock: 44.187 ± 4.97; 10 shock: 22.40 ± 2.97]. While the rate of freezing during the ISIs was not appreciably different over the period analyzed, mice in the 5 shock paradigm exhibited greater overall freezing [*F*(1,38) = 21.844, *p* < 0.0001; 5 shock: 46.07 ± 5.11, 10 shock: 19.3167 ± 2.57], stemming from elevated freezing during ISIs 3 through 5 (*p* = 0.0036, *p* = 0.0012, and *p* = 0.0016, respectively). Freezing during the last respective ISI or tone did not differ between groups (ISI: 5 shock: 53 ± 6.71; 10 shock: 48.65 ± 6.17; tone: 63.8 ± 5.23; 10 shock: 63.05 ± 4.54).

##### Extinction

Data from the first three tones were used to assess persistence of the conditioned fear after the 2-week delay. As our hypothesis centered on between-trial extinction, we analyzed the first part of the extinction trials to avoid confound of the intra-trial habituation.

#### Experiment 3 (5 shocks)

Baseline (pre-tone) freezing did not differ between the groups. Freezing increased upon tone presentation regardless of treatment [*F*(1,18) = 11.417, *p* = 0.003], but irradiated animals exhibited greater freezing to the cue (tone) than sham-irradiated mice [*F*(1,18) = 7.994, *p* = 0.011].

**Figure 4:**
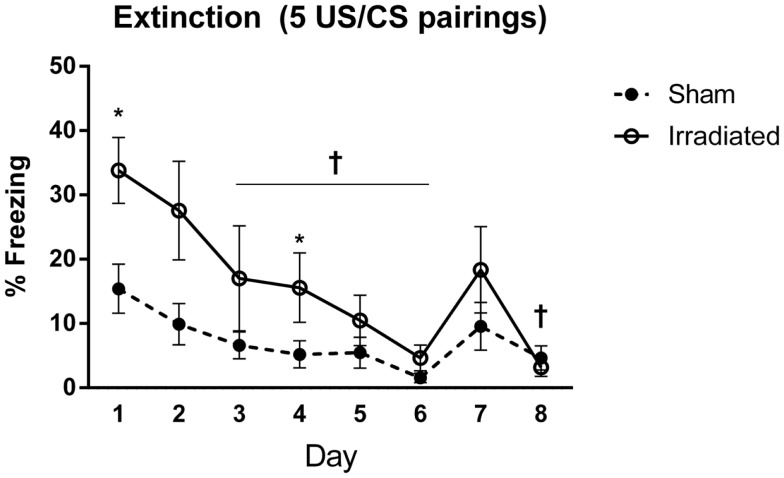
**Freezing levels during cued extinction in Experiment 1 (5 shocks)**. ^+^Sham-irradiated mice showed decreases in freezing compared to day 1 from day 3 except for day 7 (day 3, *p* = 0.018; day 4, *p* = 0.004; day 5, *p* = 0.0033; day 6, *p* = 0.0002; day 8, *p* = 0.0022). Day 7 exhibited a trend toward a decrease compared to day 1 (*p* = 0.06). Irradiated animals showed decreases in freezing compared to day 1 on day 3 (*p* = 0.0137), with decreases occurring on days 5 (*p* = 0.0015), 6 (*p* = 0.0002), 7 (*p* = 0.0104), and 8 (*p* = 0.0001). Step-wise decreases in freezing were not observed in either group. *Freezing was elevated in the irradiated mice relative to sham on days 1 (*p* = 0.008) and 4 (*p* = 0.022).

Both groups demonstrated extinction of cued fear [λ = 0.155, *F*(7,12) = 9.371, *p* < 0.0001], but this was modulated by treatment group [λ = 0.727, *F*(7,12) = 4.566, *p* = 0.011]. Both groups indicated an effect of day on freezing levels [sham-irradiated: *F*(7,63) = 9.032, *p* < 0.0001; irradiated: *F*(7,63) = 6.892, *p* < 0.0001], but decreases in freezing compared to day 1 were blunted in the irradiated group compared to the sham-irradiated mice comparing freezing at each day between the groups indicated that freezing was elevated in the irradiated mice relative to sham [λ = 0.258, *F*(8,11) = 3.956, *p* = 0.019], specifically on days 1 (*p* = 0.008) and 4 (*p* = 0.022) (Figure 4).

#### Experiment 4 (10 shocks)

Baseline (pre-tone) freezing did not differ between groups. Freezing levels increased upon tone presentation regardless of treatment [*F*(1,18) = 24.660, *p* < 0.0001] and no treatment differences were observed.

**Figure 5:**
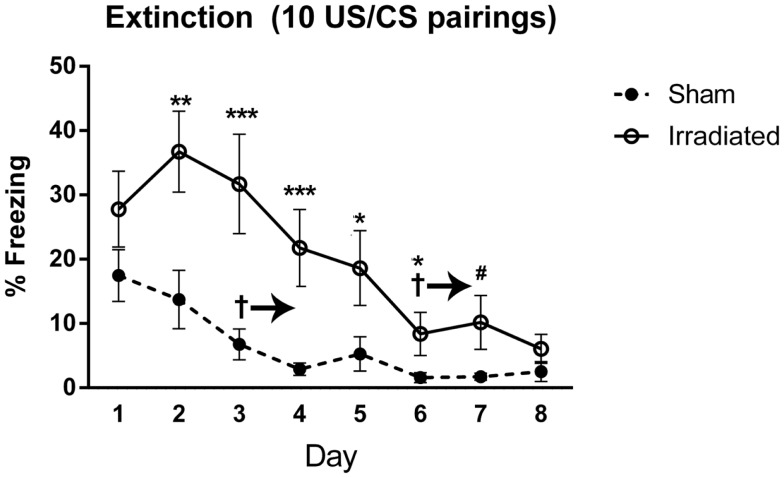
**Freezing levels during cued extinction in Experiment 2 (10 shocks)**. ^+^Sham-irradiated mice exhibited extinction by day 3 and onward (day 3, *p* = 0.019; day 4, *p* = 0.0016; day 5, *p* = 0.0026; day 6, *p* = 0.0005; day 7, *p* = 0.0007; day 8, *p* = 0.0005). Irradiated mice showed a decrease in freezing compared to day 1 by and continuing from day 6 (day 6, *p* = 0.0039; day 7, *p* = 0.0040; day 8, *p* = 0.0018). Irradiated animals exhibited greater freezing than sham-irradiated animals on days 2 (**p = 0.005), 3 (***p = 0.001), 4 (***p = 0.001), 5 (*p = 0.038), 6 (*p = 0.027), and a trend toward elevated freezing on day 7 (^#^*p* = 0.081).

Both groups demonstrated extinction of cued fear [*F*(7,126) = 20.624, *p* < 0.0001], with a trend toward an interaction with treatment group and time [*F*(7,126) = 1.782, *p* = 0.097]. Freezing levels were lower on day 3 and subsequent days as compared to day 1 (day 3 vs. 1, *p* = 0.030; day 4 vs. 1, *p* = 0.025; days 5, 6, 7, 8 vs. 1, *p* < 0.0001). Freezing levels continued to decrease, but there were no significant difference in freezing levels between directly subsequent days. Extinction was present in both groups [irradiated: λ = 0.015, *F*(7,3) = 28.406, *p* = 0.010; sham: λ = 0.040, *F*(7,3) = 10.294, *p* = 0.041], but appeared to be delayed in irradiated mice (starting from day 6, *p* = 0.0039; day 7, *p* = 0.0040; day 8, *p* = 0.0018), compared to day 3 in sham-irradiated mice (day 3 and onward: day 3, *p* = 0.019; day 4, *p* = 0.0016; day 5, *p* = 0.0026; day 6, *p* = 0.0005; day 7, *p* = 0.0007; day 8, *p* = 0.0005). Irradiated animals exhibited greater freezing than sham-irradiated animals on days 2 (*p* = 0.005), 3 (*p* = 0.001), 4 (*p* = 0.001), 5 (*p* = 0.038), 6 (*p* = 0.027), and a trend toward elevated freezing on day 7 (*p* = 0.081) (Figure 5). There was also a between-subjects effect of treatment, with irradiated animals spending more time freezing overall than sham-irradiated mice [*F*(1,18) = 11.261, *p* = 0.004].

In both cued experiments, freezing in the irradiated animals was elevated on day 1 compared to the sham animals [*F*(1,18) = 7.191, *p* = 0.15]. To compare extinction without the influence of different starting freezing levels, extinction levels in 10 shock-sham-irradiated and 5 shock-irradiated mice were compared (Figure 3C), as they were individually at parity. There was an effect of day [*F*(7,126) = 17.847, *p* < 0.0001], but no interaction with treatment. Overall freezing levels were still higher in irradiated than sham-irradiated mice [between-subjects effect of treatment, irradiated animals > sham, *F*(1,18) = 5.306, *p* = 0.033]. This was due to elevated freezing overall, and not elevated freezing specifically at any 1 day (Figure 3C).

##### Measures of anxiety in the elevated zero maze

**Figure 6:**
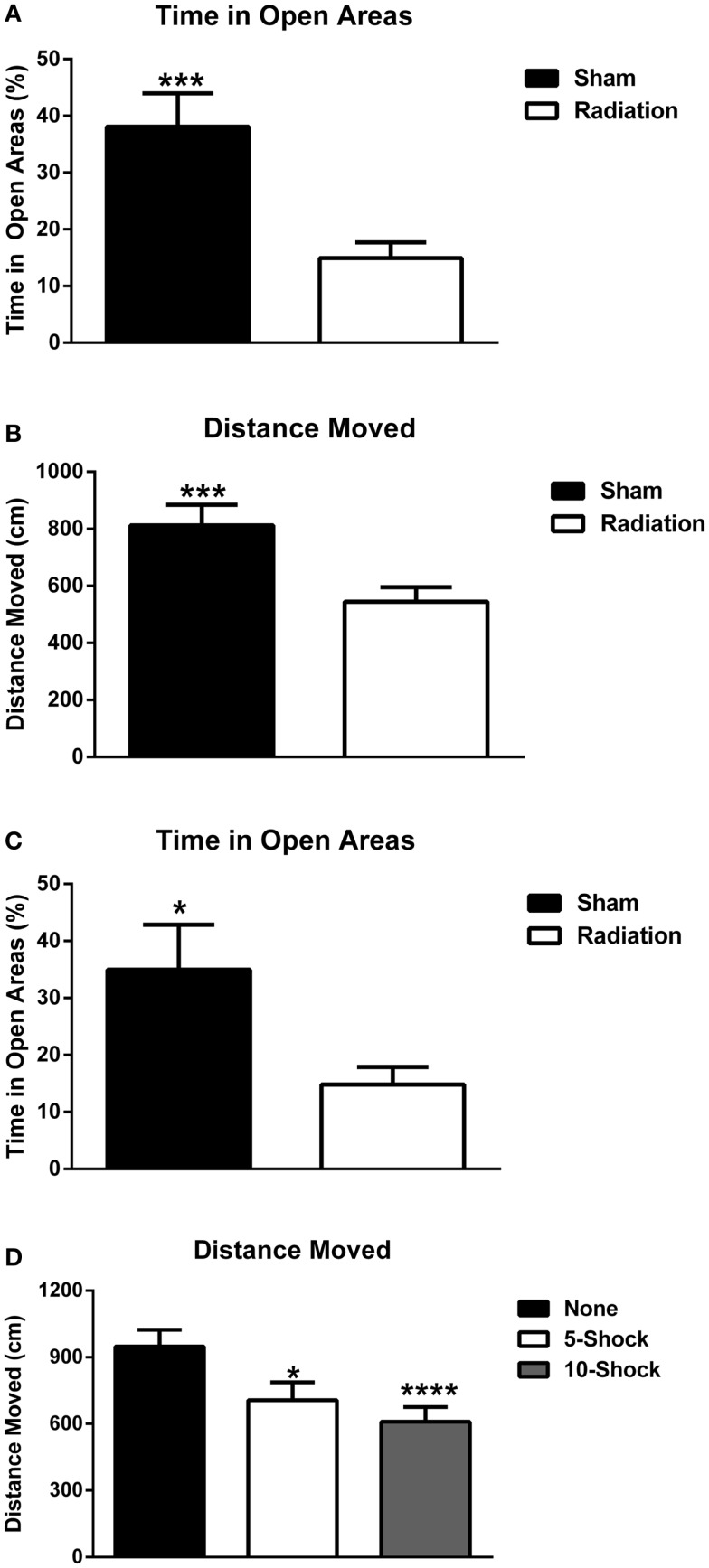
**(A)** Effect of irradiation on measures of anxiety of mice trained and tested for cued fear conditioning in the elevated zero maze. Irradiated mice showed enhanced anxiety levels and spent less time in the more anxiety-provoking open areas. ***p < 0.001. **(B)** Effects of irradiation on activity levels of mice trained and tested for cued fear conditioning in the elevated zero maze. Irradiated mice moved less than sham-irradiated mice. ***p = 0.002. **(C)** Effects of irradiation on measures of anxiety of a combined group of behaviorally naïve mice and mice trained and tested for cued fear conditioning in the elevated zero maze. Irradiated mice showed enhanced anxiety levels and spent less time in the more anxiety-provoking open areas of the maze. ***p < 0.0001. **(D)** Effects of number of shocks on activity levels of mice in the elevated zero maze. Mice that had received shocks showed lower activity levels than those that did not and this was more pronounced in mice that had received 10 shocks than those that had received 5 shocks. *p = 0.001, ****p < 0.0001.

Animals part of the cued fear conditioning experiment were analyzed for measures of anxiety using experimental group (5 or 10 shock) and radiation exposure (4 Gy or sham) as between-subject groups. Irradiated animals spent significantly less time in the open areas of the elevated zero maze [Figure 6A; *F*(1,36) = 13.147, *p* < 0.001] regardless of whether they received 5 or 10 shocks 22 days prior (see Figure S1 in Supplementary Material for 5 and 10 shock groups displayed separately. ANOVA was used for the analysis to assess whether the effects of irradiation on measures of anxiety was dependent on the number of shocks.). Irradiated animals also exhibited reduced exploratory behavior, as measured by distance moved [Figure 6B; *F*(1,36) = 11.725, *p* = 0.002] and nose-pokes into the open areas of the elevated zero maze (5 shock sham-irradiated: 5.00 ± 1.25; 10 shock-irradiated: 2.40 ± 0.733; 5 shock irradiated: 3.90 ± 0.72; 10 shock irradiated: 4.70 ± 1.33, see also Figure S1 in Supplementary Material). This can be a confounding factor. However, while increasing training increased freezing in the irradiated groups, this “training-dose” effect was not observed for measures of locomotor function. Although this does not rule out the contribution of a locomotor deficit, the lack of a group difference in locomotion parallel to increased freezing suggests that the pronounced freezing is due to other factors such as recall of the fear memory.

In Experiment 5, a separate group of 20 animals who did not receive fear conditioning were irradiated or sham-irradiated (*n* = 10 mice/group) and evaluated for anxiety-like phenotypes in the elevated zero maze, 12 days after irradiation (the time point when fear conditioning would have begun). This was to assess for potential differences in anxiety at the onset of extinction trials due to radiation alone. Irradiated animals spent significantly less % time in the open areas of the elevated zero maze [sham-irradiated: 34.92 ± 7.95; irradiated, 14.82 ± 3.08; *F*(1,17) = 31.310, *p* < 0.0001]. Irradiated animals exhibited spent less time exploring compared to controls, moving less distance in the maze [sham-irradiated: 1161.54 ± 80.73 cm; irradiated: 712.05 ± 73.08 cm; *F*(1,17) = 16.755, *p* = 0.0008], though nose-pokes into the open areas of the elevated zero maze did not differ between the groups (sham-irradiated: 3.2 ± 0.79; irradiated: 3.56 ± 0.78).

To evaluate the contribution of fear conditioning-related anxiety, data from Experiments 3, 4, and 5 were analyzed together. The data from all mice were combined and analyzed together, with radiation dose and shock paradigm (none, 5, and 10 shocks) as between-subject variables. An effect of radiation, but not fear conditioning, indicated that irradiated animals overall spent less time in the open areas of the elevated zero maze [Figure 6C; *F*(1,53) = 18.874, *p* < 0.0001]. Distance moved was affected by both radiation exposure [*F*(1,53) = 25.549, *p* < 0.0001] and fear conditioning [*F*(2,53) = 7.765, *p* = 0.001]. Irradiated animals moved less than sham-irradiated controls (573.51 ± 44.92 vs. 928.71 ± 62.41 cm, *p* < 0.0001). In addition, animals that were not tested for fear conditioning moved more (948.62 ± 75.08 cm) than mice that had received fear conditioning training involving 5 (718.19 ± 76.96 cm, *p* = 0.013) or 10 shocks (605.28 ± 63.28, *p* < 0.0001) (Figure 6D). As there was an overall effect of radiation but no interaction between effects of radiation and number of shocks, sham-irradiated and irradiated mice were collapsed for displaying the data in Figure 6D. No differences in nose-poking behavior were observed.

### Hippocampal MAP-2 levels (Experiments 1 and 2)

**Figure 7:**
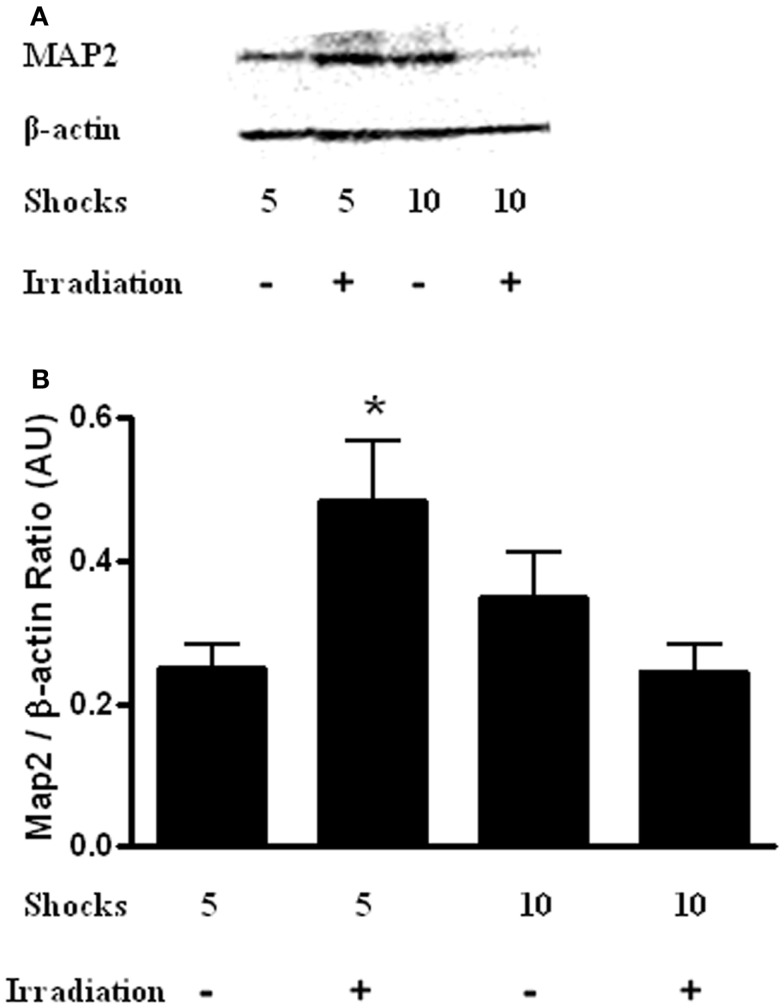
**(A)** Representative western blot of hippocampal MAP-2 levels in sham-irradiated and irradiated mice that received 5 or 10 shocks during training. **(B)** There was a radiation × shock interaction with higher hippocampal MAP-2 levels in irradiated mice that received 5 shocks than is sham-irradiated mice that received 5 shocks and irradiated mice received 10 shocks. *p < 0.05 vs. sham-irradiated mice that received 5 shocks and irradiated mice received 10 shocks.

Finally, hippocampal levels of MAP-2 were determined. There was a radiation × shock interaction [Figure 7; *F*(3,14) = 9.565, *p* = 0.10]. MAP-2 levels were higher in irradiated mice that received five shocks than sham-irradiated mice that received five shocks (*t* = 2.932, *p* = 0.0326). In addition, MAP-2 levels were higher in irradiated mice that received 5 shocks than irradiated mice that received 10 shocks (*t* = 2.876, *p* = 0.0348).

## Discussion

The data of the current study show that in mice exposure to whole-body irradiation, 24 h following training is associated with reduced daily increases in body weights over the 22 days of the study. This is consistent with exposure to radiation in human studies (Johnson et al., 1982). It is important to note that contextual recall, extinction, and anxiety tests began after weight-changes had normalized. While we did not monitor food intake (animals were group housed, with irradiated and non-irradiated animals housed together), we did not observe any gross ill effects while monitoring and checking on animals during the periods preceding extinction trials, or signs of diarrhea prior to or during the extinction trials. Based on the relatively low dose of radiation used in this study, we did not anticipate diarrhea. For example, Saha et al. (2012) used 8.4–10.4 Gy in their study and Boothm et al. (2012) saw diarrhea in mice irradiated with 13 Gy or more in their 2013 study. Exposure to whole-body irradiation 24 h following contextual fear conditioning training resulted in greater freezing levels and aberrant extinction 2 weeks after training. This general effect was observed even after increasing the intensity of the training protocol to control for the lower levels of freezing in the control group. This is likely due in part to impaired or disturbed memory processes. Mice that received irradiation and the less intense training paradigm (5 shocks) and those who received no irradiation and the more intense paradigm (10 shocks) showed comparable recall of the fear memory on the first day of re-exposure to the context (2 weeks after training), yet extinction of the contextual fear memory in the irradiated group was impaired relative to the sham group. Especially, as measures of anxiety and contextual and cued freezing are both increased 2 weeks after irradiation, matching the freezing levels on the first day of extinction as a separate analysis is important for the interpretation of the data and identification of deficit in extinction learning. Similar to contextual freezing levels, cued freezing levels 2 weeks after training were also higher in irradiated than sham-irradiated mice. In contrast to contextual freezing levels, cued freezing levels were even higher in irradiated mice receiving 5 shocks during training than sham-irradiated mice receiving 10 shocks during training, suggesting a prolonged deficit in recall of the conditioned fear that could not be overcome by additional training. Interestingly, anxiety was elevated in irradiated animals tested 2 weeks after irradiation in mice that were trained in cued fear conditioning, an effect that was conserved in a behaviorally naïve non-fear conditioned cohort. In summary, whole-body irradiation elevated contextual and cued fear memory recall across all paradigms. Differences in recall of contextual fear could be obviated by over-training the sham-irradiated animals, though this enhanced training did not replicate the pattern of extinction observed in the irradiated animals – which was still comparatively delayed. In the cued fear experiments, freezing levels remained elevated after similar comparison, though effects of radiation on extinction of cued fear was less pronounced compared to effects of radiation on extinction of contextual fear. These data suggest enhanced sensitivity of hippocampal-dependent memory processes and point to perturbations in different memory processes. Given that radiation induced a prolonged increase in anxiety-like behavior even 2-weeks after training, anxiety may modulate the observed effects. Nevertheless, the more profound impairment of extinction of contextual fear – even after elevating sham-irradiated animals to parity on day 1 – suggests that the hippocampus might be particularly susceptible to this radiation effect. While we believe that there is a strong anxiety component, the impairment in extinction when comparing the groups that showed parity on day 1 of extinction was not found in cued animals.

Fear conditioning, especially the presence or absence of inhibition or extinction of learned fear (Cannistraro and Rausch, 2003), is used to study recurring and re-experiencing symptoms of post-traumatic stress disorder (PTSD) in both humans and animal models (Siegmund and Wotjak, 2006; Olsen et al., 2012). These symptoms may be related to a failure of extinction learning or a failure to modify or acquire new associations to contextual stimuli (Charney et al., 1993; Corcoran et al., 2005). The prevalence of PTSD and heterogeneous response to the trauma suggests the involvement of environmental risk factors (Kessler et al., 1995; Breslau et al., 1998) and associations between intensity and number of traumatic events (Sledjeski et al., 2008). The data of the current study indicate that whole-body radiation exposure might be such an environmental risk factor, and therefore has implications for emergency responders, such as those attending to the Fukushima nuclear accident in 2011 or persons exposed during a nuclear conflict. In fact, human radiation exposure related to accidents or subsequent cleanup efforts at nuclear power plants are consistent with a post-traumatic stress response and increased PTSD risk following whole-body irradiation (House et al., 1992; Havenaar et al., 1997; Rahu et al., 2006; Shigemura et al., 2012). In addition to nuclear accidents, potential radiation exposure is also pertinent to military missions and dirty bomb scenarios (Chin, 2007; Giesecke et al., 2012; Obenaus et al., 2012).

A limitation of the human data is that it is often hard to exclude potential effects of the stress associated with a nuclear accident and the subsequent refugee scenario or even the threat of a nuclear accident occurring (Korol et al., 1999) in the absence of any radiation exposure. The continued distress of dogs abandoned following the Fukushima accident (Nagasawa et al., 2012) is an example of long-term alterations in stress responses that complicate evaluating the effects of radiation on the stress response in these real life situations and highlight the importance of environmentally controlled animal experiments. Enhanced anxiety levels and environmental stressors not directly related to the initial trauma, such as discrimination in the context of a nuclear accident (Shigemura et al., 2012) or insufficient societal support and societal rejection in the context of war veterans (Fontana and Rosenheck, 1994), are associated with PTSDs. Two weeks after irradiation, there were enhanced anxiety levels in the mice, also in the absence of training or testing for fear conditioning. These enhanced anxiety levels could have contributed to the enhanced fear memory and reduced extinction seen in the irradiated mice. This combination of enhanced anxiety levels and enhanced conditioned fear and reduced extinction is also seen in often-used animal models of PTSD. In the single prolonged stress (SPS) PTSD model, there are enhanced anxiety levels, enhanced contextual freezing, and impaired extinction of the fear memory (Yamamoto et al., 2009). Similarly, in the psychosocial animal model of PTSD, there are increased anxiety levels and enhanced fear memory 3 weeks after the last exposure (Zoladz et al., 2012).

Compared to paradigm-matched sham-irradiated mice, contextual freezing 2 weeks after training was higher and subsequent extinction was more delayed in mice that received 5 than 10 shocks during training. This effect of the number of shocks is consistent with the association of the number of traumatic events and the risk of developing PTSD seen in humans (Sledjeski et al., 2008).

Anxiety, fear memory, and extinction are closely linked (Herry et al., 2010), involving neuronal circuits in the amygdala, prefrontal cortex, hippocampus, and brain stem. However, fear learning, memory, and extinction, might be regulated by subregions distinct from those regulating anxiety levels. For example, using an optogenetic approach, granule cells in the dorsal dentate gyrus controlled encoding, but not retrieval, of contextual fear conditioning, while granule cells in the ventral dentate gyrus regulated anxiety levels (Fournier and Duman, 2013; Kheirbek et al., 2013). Besides regulation of anxiety levels, the ventral hippocampus is also important for gating of fear after extinction (Bouton, 2002; Hobbin et al., 2006). Inactivation of the ventral hippocampus resulted in a return of fear responses in extinguished, but not conditioned, animals (Sotres-Bayton et al., 2012). In contrast, inactivation of the basolateral amygdala reduced conditioned responses, and inactivation of the ventral hippocampus and basolateral amygdala had distinct effects on neuronal activity in the prelimbic cortex, part of the prefrontal cortex and important for the expression of conditioned fear (Sotres-Bayton et al., 2012). While memory and extinction of conditioned fear are affected by enhanced anxiety levels, enhanced anxiety levels cannot fully account for the effects of whole-body irradiation on fear memory and extinction. While fear memory was enhanced and subsequent extinction reduced in irradiated mice that had received 10 shocks during training as compared to those had received 5 shocks during training, there were no differences in measures of anxiety in these two groups of irradiated mice. The differences observed between effects of radiation on hippocampal-dependent and -independent fear recall and extinction suggest that specific memory processes are not affected in a uniform manner.

Irradiated mice that received five shocks during trained showed increased hippocampal MAP-2 levels as compared to sham-irradiated mice. Consistent with these data, MAP-2 levels in the dentate gyrus were increased following brain only ^56^Fe irradiation (Villasana et al., 2013). In this study, the mice received two tone-shock pairings during fear conditioning training, performance was assessed in additional tests, and the brains were analyzed 3 months following irradiation. The radiation-induced increase in MAP-2 levels might be part of a compensatory change. Increased MAP-2 levels have been also seen in the hippocampus of non-human primates (Haley et al., 2010) and brains of aged mice (Benice et al., 2006). Interestingly, these radiation-induced increased hippocampal MAP-2 levels were not seen in irradiated mice that received 10 shocks. These data suggest that the additional aversive stimuli during training might prevent this increase. The discrepancy in the pattern of acquisition and extinction and hippocampal MAP-2 levels suggests that changes in this marker of dendritic morphology cannot explain the effects of irradiation or the number of shock on cognitive function 2 weeks after irradiation. It is important to note that brain tissue was taken following extinction, and this should not be construed to represent acute changes following radiation.

Components of fear conditioning involve hippocampal neurogenesis (Kheirbeck et al., 2012; Fitzsimons et al., 2013; Pan et al., 2013). Selective knockdown of the glucocorticoid receptor in newborn neurons in the adult hippocampus accelerated their neuronal differentiation and migration, increased the number of mature spines and mossy fiber boutons, and impaired contextual fear conditioning (Fitzsimons et al., 2013). As hippocampal neurogenesis is strongly reduced following irradiation (Raber et al., 2004a,b), it might contribute to the enhanced fear memory, although 2 weeks seems a relatively short period for newborn cells to become functionally integrated into a neuronal network. Alternative mechanisms underlying these radiation effects might involve L-type voltage-dependent calcium channels shown to modulate contextual fear conditioning (McKinney et al., 2008) or epigenetic mechanisms (Heinzelmann and Gill, 2013) and especially DNA methylation and hydroxymethylation, as mice deficient in Tet1, a member of the 10–11 translocation (Tet) family, which catalyzes the oxidation of 5-methylcytosine to 5-hydroxymethylcytosine and promotes DNA demethylation, were unable to extinguish contextual fear (Rudenko et al., 2012).

Recently, we reported enhanced synaptic plasticity in the CA1 region of the hippocampus and enhanced contextual fear memory in mice trained and tested for contextual fear memory 3 months following ^28^Si irradiation (Raber et al., 2014). Therefore, we initially focused on hippocampal MAP-2 levels in the current study. However, additional brain regions such as the medial prefrontal cortex, anterior cingulate cortex, and basolateral and central nuclei of the amygdala might be involved in these radiation effects. Therefore, it will be important to determine the anatomical specificity of post-training irradiation effects on fear memory in future studies. For example, based on the time point for memory recall used in the current study, remote memory might be involved. For remote memory, the anterior cingulate cortex can play a very important compensatory role for hippocampal dysfunction (Goshen et al., 2011). This compensation might especially happen at a younger age and future studies are warranted to determine whether the post-training radiation effects are age-dependent or not. The age of the animals can also have a strong impact on the efficacy of extinction training.

In the current study, we see enhanced contextual fear conditioning following X-ray irradiation. Interestingly, contextual fear conditioning is also enhanced 3 months following ^28^Si irradiation (Raber et al., 2014). As this cognitive enhancing effect was not seen following ^56^Fe irradiation (Villasana et al., 2010), the type of radiation might be important here. It is also important to note that in the Villasana et al. (2010), study another anxiety test was used (the open field), which might differ in terms of sensitivity to detect effects of irradiation on measures of anxiety. Also, as the testing of the mice in the current study was 2 weeks after irradiation but in the Villasana et al. (2010) study was 3 months following irradiation, it is conceivable that measures of anxiety were increased at earlier time points following irradiation but returned to levels comparable to those in sham-irradiated mice at 3 months. Finally, it should be noted that for ^56^Fe and ^28^Si irradiation studies mice were shipped from JAX laboratories to Brookhaven National Laboratories, Long Island, NY, USA and subsequently to OHSU following irradiation while the X-ray irradiated mice used in the current study were only shipped from JAX labs to OHSU prior to irradiation. Therefore, it is hard to compare these studies.

As whole-body irradiation was used in the current study, it is not possible to distinguish between direct effects of irradiation on the hippocampus and indirect effects of irradiation on the hippocampus or other brain areas via effects of irradiation on peripheral areas of the body. Future studies using brain only irradiation could be used to address this. Although neuronal progenitor cells are sensitive to irradiation, it is difficult to determine whether neurogenesis plays a role in the radiation effects seen in the current study. In Ko et al. (2009), neurogenesis was specifically ablated prior to training, and therefore circuit mechanisms present during the formation of the fear memory may have involved some compensatory mechanisms that did not affect extinction. Also, irradiation took place 3 months prior to training and extinction. Because only sparse populations of neural stem cells are proliferating at any one time, changes to proliferating cell populations may have already compensated by that time, obviating or reducing the consequence on fear conditioning. Additionally, when MAM was used in that study to ablate neurogenesis more proximal to the conditioning/extinction paradigm, there was no reduction in the incorporation of BrDU. Based on the generally accepted time line required for new neurons (about 4 weeks) to become functionally integrated in the hippocampus, it is unlikely neurogenesis played a role.

In summary, the data of the current study show enhanced fear memory, reduced extinction, and enhanced anxiety levels in mice that received whole-body irradiation following acquisition of fear conditioning 2 weeks earlier. These behavioral and cognitive changes are pertinent to radiation exposure as part of a nuclear accident, military mission, or dirty bomb scenario, and reminiscent to symptoms seen in PTSD, a common and debilitating anxiety disorder (frequently comorbid with other mental disorders). Future efforts are warranted to determine the molecular mechanisms underlying these post-training radiation effects.

## Author Contributions

Reid H. J. Olsen: experimental design; acquisition and analysis of data, interpretation of the data, and input to the manuscript. Tessa Marzulla: acquisition and analysis of data, interpretation of the data, and input to the manuscript. Jacob Raber: experimental design, interpretation of the data, and input to the manuscript.

## Conflict of Interest Statement

The authors declare that the research was conducted in the absence of any commercial or financial relationships that could be construed as a potential conflict of interest.
