# Supplementary material for: Impairment in Extinction of Contextual and Cued Fear Following Post-Training Whole-Body Irradiation
Source: Front Behav Neurosci. 2014 Jul 2;8:231. doi: 10.3389/fnbeh.2014.00231 (PMC4078460; doi:10.3389/fnbeh.2014.00231)
Supplement: Supplementary file 1 [file Data_Sheet1.PDF]

Supplementary Table 1. Motion during shock in Experiment 1 (5 shocks)

**Dose**	**Motion During Shock**[1]				
	**Shock 1**	**Shock 2**	**Shock 3**	**Shock 4**	**Shock 5**
**0**	556.44 ± 38.88	562.81 ± 54.74	608.69 ± 50.92	615.16 ± 45.78	707.62 ± 87.98
**4 Gy**	496.54 ± 44.24	490.79 ± 41.15	617.82 ± 45.38	539.00 ± 64.23	701.50 ± 83.57

[1]A repeated measures ANOVA with treatment as a between-subject variable revealed an effect of shock ($F(4,72) = 9.183$, $p < 0.0001$), but no interaction with treatment. Step-wise increases in motion during shock were found during shock 3 (as compared to shock 2, $p = 0.043$) and shock 5 (as compared to shock 4, $p = 0.043$).

Supplementary Table 2. Motion during shock in Experiment 2 (10 shocks)

	Motion During Shock									
**Dose**	**Shock 1**	**Shock 2**	**Shock 3**	**Shock 4**	**Shock 5**	**Shock 6**	**Shock 7**	**Shock 8**	**Shock 9**	**Shock 10**
**0**	534.52 ± 54.77	528.93 ± 42.22	594.04 ± 70.53	662.26 ± 75.16	560.32 ± 57.88	634.96 ± 36.00	537.28 ± 92.87	608.45 ± 64.20	607.90 ± 80.45	610.16 ± 79.80
**4 Gy**	434.86 ± 46.63	491.81 ± 50.72	656.89 ± 71.49	553.17 ± 74.94	578.26 ± 66.82	609.85 ± 50.09	679.21 ± 105.34	578.92 ± 77.52	552.08 ± 88.45	603.41 ± 57.49

A repeated measures ANOVA with treatment as a between-subject variable highlighted a trend toward an effect of shock ($F(9,162) = 1.773$, $p = 0.077$), but no interaction with treatment. No differences between temporally adjacent shocks were identified.

Supplementary Table 3. Freezing levels during contextual extinction in Experiment 1 (5 shocks) [1].

Dose	Day 1[3]	Day 2[3]	Day 3	Day 4[2]	Day 5[3]	Day 6[3]	Day 7	Day 8	Day 9 Baseline	Reinstatement[4]	Day 10
**0**	4.46 ± 1.44	1.51 ± 0.51	2.36 ± 0.75	1.18 ± 0.42	1.38 ± 0.55	0.96 ± 0.47	0.63 ± 0.26	0.99 ± 0.56	1.03 ± 0.83	1.16 ± 0.19	1.29 ± 0.67
**4 Gy**	12.74 ± 3.00	12.23 ± 0.39	9.28 ± 3.48	3.84 ± 1.61	5.67 ± 1.31	3.16 ± 0.96	1.09 ± 0.54	2.17 ± 0.81	1.59 ± 1.00	7.87 ± 2.58	2.77 ± 1.90

[1] Freezing levels were calculated during the first five minutes of a trial.

[2] Reduced freezing levels, relative to those seen on Day 1, began on day 4 for sham animals (day 4 vs day 1, $p = 0.0139$) and continued through day 8 (day 5 vs day 1, $p = 0.0147$; day 6 vs day 1, $p = 0.0056$; day 7 vs day 1, $p = 0.0029$; day 8 vs day 1, $p = 0.0061$). Extinction began on day 4 for irradiated animals (day 4, p = 0.001), and continued from day 6 on (day 6, $p = 0.0018$; day 7, $p < 0.0001$; day 8, $p = 0.0003$). Stepwise extinction did not occur in either group.

[3] Irradiated mice exhibited greater freezing than sham-irradiated mice on days 1 ($p = 0.021$, 2 ($p = 0.010$), 5 ($p = 0.003$) and 6 ($p = 0.0028$)

[4] Sham-irradiated mice exhibited a trend toward a significant increase in freezing following the reinstatement shock ($p = 0.084$). Irradiated mice exhibited a significant increase in freezing immediately following the reinstatement ($p = 0.0273$). Freezing levels following reinstatement were greater in the irradiated group ($p = 0.019$).

Supplementary Table 4. Freezing levels during contextual extinction in Experiment 2 (10 shocks).

Dose	Day 1	Day 2[3]	Day 3[1,2,3]	Day 4[3]	Day 5	Day 6[2]	Day 7	Day 8	Day 9 Baseline	Reinstatement[4]	Day 10[5]
**0**	13.86 ± 4.55	4.18 ± 0.94	0.38 ± 0.12	1.25 ± 0.55	3.92 ± 1.31	1.29 ± 0.69	0.96 ± 0.41	1.37 ± 0.50	0.71 ± 0.47	6.03 ± 1.91	2.70 ± 0.98
**4Gy**	29.38 ± 6.64	17.97 ± 6.57	7.67 ± 1.80	11.97 ± 3.57	6.92 ± 1.85	1.91 ± 0.53	1.77 ± 0.83	2.93 ± 1.04	1.28 ± 0.66	7.22 ± 3.02	8.70 ± 2.71

[1] Reduced freezing levels, relative to those on day 1, began on day 3 for sham animals (day 3 vs day 1, $p = 0.0002$, day 4, $p = 0.0007$; day 5, $p = 0.0149$; day 6, $p = 0.0006$, day 7, $p = 0.0005$; day 8, $p = 0.0009$). Within the irradiated group, freezing levels reduced by day 3 and persisted throughout the experiment (day 3, $p = 0.0029$; day 4, $p = 0.0111$; day 5, $p = 0.0023$; day 6, $p < 0.0001$; day 7, $p < 0.0001$; day 8, $p < 0.0001$).
[2] Within the sham-irradiated group, a stepwise decease in freezing occurred only between days 2 and 3 ($p = 0.0004$). Within the irradiated group, a stepwise decrease in freezing occurred only between days 6 and 7 ($p = 0.0269$).
[3]Irradiated mice exhibited greater freezing than sham-irradiated mice on days 2 ($p = 0.005$) 3 ($p < 0.0001$), and 4 ($p = 0.002$). Irradiated mice exhibited a trend toward greater freezing on day 1 ($p = 0.083$).
[4]Both groups exhibited an increase in freezing following the reinstatement shock (Sham-irradiated $p = 0.0156$; Irradiated $p = 0.0234$).

[5]Irradiated mice exhibited a trend toward an increase in freezing 24 hours after the reinstatement of the fear memory ($p = 0.084$).

Table 5. Motion during shock in Experiment 2 (10 shocks)

Dose	Motion During Shock[1]				
	**Shock 1**	**Shock 2**	**Shock 3**	**Shock 4**	**Shock 5**
**0**	796.00 ± 69.20	811.00 ± 67.92	828.50 ± 72.02	835.50 ± 110.65	993.50 ± 78.54
**4 Gy**	694.80 ± 59.66	747.60 ± 63.47	846.90 ± 83.15	888.20 ± 90.40	970.10 ± 86.07

The average motion during the shocks did not differ between the groups. An effect of shock-order was indicated ($F(4,72) = 6.047$, $p < 0.0001$) with motion during shock 5 being statistically greater than shocks 1 ($p = 0.003$)and 2 ($p = 0.007$).

Table 6. Motion during shock in Experiment 2 (10 shocks).

	Motion During Shock									
**Dose**	**Shock 1**	**Shock 2**	**Shock 3**	**Shock 4**	**Shock 5**	**Shock 6**	**Shock 7**	**Shock 8**	**Shock 9**	**Shock 10**
**0**	681.80 ± 45.74	815 ± 59.97	744.80 ± 62.46	753.70 ± 63.67	925.80 ± 52.59	930.75 ± 89.00	821.96 ± 79.13	867.16 ± 91.49	815.00 ± 75.51	847.46 ± 78.27
**4 Gy**	789.40 ± 49.19	765.00 ± 52.41	828.80 ± 77.30	915.40 ± 78.28	739.5 ± 59.53	851.02 ± 36.64	851.11 ± 58.91	912.76 ± 82.21	914.00 ± 74.43	807.57 ± 80.24

Increases in motion during shock were not significantly different.

Supplementary Fig. 1A. Effect of irradiation on measures of anxiety of mice trained and tested for cued fear conditioning, involving 5 or 10 shocks, in the elevated zero maze. Irradiated mice showed enhanced anxiety levels and spent less time in the more anxiety-provoking open areas. **Fig. 1B.** Effects of irradiation on activity levels of mice trained and tested for cued fear conditioning, involving 5 or 10 shocks, in the elevated zero maze. Irradiated mice moved less than sham-irradiated mice.

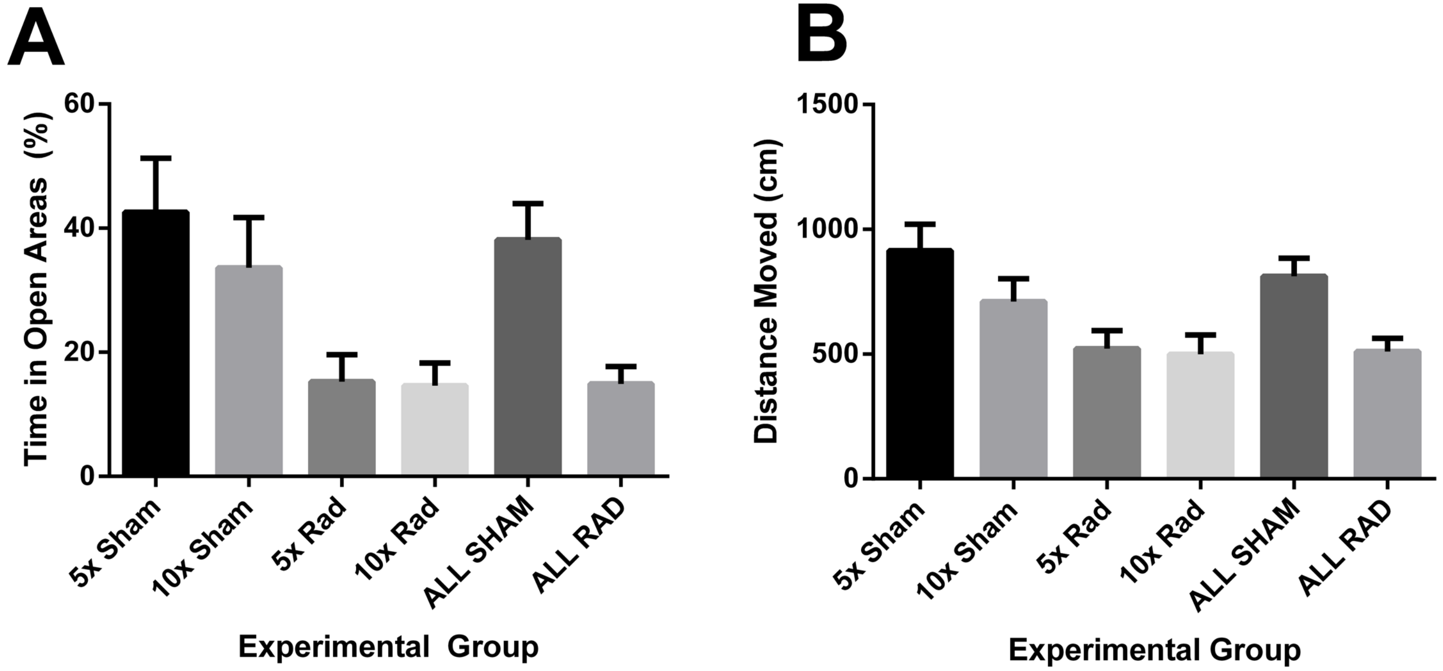
A
Time in Open Areas (%)
60
40
20
0
5x Sham
10x Sham
5x Rad
10x Rad
ALL SHAM
ALL RAD
Experimental Group
B
Distance Moved (cm)
1500
1000
500
0
5x Sham
10x Sham
5x Rad
10x Rad
ALL SHAM
ALL RAD
Experimental Group
